# Mangosteen Pericarp Extract Mitigates Diquat-Induced Hepatic Oxidative Stress by NRF2/HO-1 Activation, Intestinal Barrier Integrity Restoration, and Gut Microbiota Modulation

**DOI:** 10.3390/antiox14091045

**Published:** 2025-08-25

**Authors:** Weichen Huang, Yujie Lv, Chenhao Zou, Chaoyue Ge, Shenao Zhan, Xinyu Shen, Lianchi Wu, Xiaoxu Wang, Hongmeng Yuan, Gang Lin, Dongyou Yu, Bing Liu

**Affiliations:** 1 Zhejiang Key Laboratory of Nutrition and Breeding for High-Quality Animal Products, College of Animal Sciences, Zhejiang University, Hangzhou 310058, China; 22317098@zju.edu.cn (W.H.); 22217093@zju.edu.cn (Y.L.); chenhao.zou@zju.edu.cn (C.Z.); 22117078@zju.edu.cn (C.G.); 22317012@zju.edu.cn (S.Z.); 22317063@zju.edu.cn (X.S.); 22217068@zju.edu.cn (L.W.); 22417088@zju.edu.cn (X.W.); 22417094@zju.edu.cn (H.Y.); 2 ZJU-Xinchang Joint Innovation Centre (TianMu Laboratory), Gaochuang Hi-Tech Park, Shaoxing 312500, China; 3 Hainan Institute, Zhejiang University, Sanya 572000, China; 4 Institute of Quality Standards and Testing Technology for Agricultural Products, Chinese Academy of Agricultural Sciences, Beijing 100081, China; bluewhalebell@163.com

**Keywords:** mangosteen pericarp extract, oxidative stress, nutritional regulation, gut microbiota, poultry, fecal microbiota transplantation, antioxidant mechanism

## Abstract

Poultry production exposes birds to diverse environmental and physiological stressors that disrupt redox balance, impair gut–liver axis function, and undermine health and productivity. This study investigated the hepatoprotective and antioxidative effects of mangosteen pericarp extract (MPE) in an experimental model of diquat-induced oxidative stress in laying hens. A total of 270 Hy-Line White laying hens were randomly assigned to three groups: control group (CON), diquat-challenged group (DQ), and MEP intervention with diquat-challenged group (MQ), with six replicates of 15 birds each. The results showed that MPE supplementation effectively mitigated the hepatic oxidative damage caused by diquat, as evidenced by the increased ALT and AST activity, improved lipid metabolism, and reduced hepatic fibrosis. Mechanistically, MPE activated the NRF2/HO-1 antioxidant pathway, thus enhancing the liver’s ability to counteract ROS-induced damage and reducing lipid droplet accumulation in liver tissue. MPE supplementation restored intestinal barrier integrity by upregulating tight junction protein expression (Occludin-1 and ZO-1), enhancing MUC-2 expression, and thereby decreasing gut microbiota-derived LPS transferring from the intestine. Additionally, MPE also modulated gut microbiota composition by enriching beneficial bacterial genera such as *Lactobacillus* and *Ruminococcus* while suppressing the growth of potentially harmful taxa (e.g., *Bacteroidales* and *UCG-010*). Fecal microbiota transplantation (FMT) from MPE-treated donors into diquat-exposed recipients reproduced these beneficial effects, further highlighting the role of gut microbiota modulation in mediating MPE’s systemic protective actions. Together, these findings demonstrated that MPE alleviated DQ-induced liver injury and oxidative stress through a combination of antioxidant activity, protection of intestinal barrier function, and modulation of gut microbiota, positioning MPE as a promising natural strategy for mitigating oxidative stress-related liver damage by regulating the gut microbiota and gut–liver axis in poultry.

## 1. Introduction

In modern poultry and livestock production, animals are frequently exposed to a wide array of environmental and physiological stressors—such as heat stress, mycotoxins, heavy metals, pathogenic infections, and high-density housing—that disrupt redox homeostasis and trigger oxidative stress [1,2,3]. The resulting oxidative imbalance impairs feed intake, suppresses growth, and provokes intestinal inflammation, leading to compromised development and overall production efficiency [4,5]. Excessive generation of reactive oxygen species (ROS), when not sufficiently neutralized by endogenous antioxidants, leads to cellular and organelle damage, particularly in high metabolic organs like the liver and intestine [6,7]. In laying hens, oxidative stress has been shown to impair hepatic lipid metabolism, weaken intestinal barrier integrity, reduce antioxidant enzyme activities, and disrupt gut microbiota composition, ultimately compromising egg production and quality [8,9]. Given the central role of oxidative stress in mediating gut–liver axis dysfunction and performance decline, identifying effective nutritional strategies to counteract redox imbalance is of critical importance for sustaining poultry health and productivity.

Mangosteen (*Garcinia mangostana*) is a tropical evergreen tree native to regions around the Indian Ocean, valued both for its edible fruit and its long history of traditional medicinal use [10,11,12]. Mangosteen pericarp is often discarded as agricultural waste, but mangosteen pericarp extract (MPE) is rich in phenolics—tannins, flavonoids, and especially xanthone—with diverse bioactivities such as antioxidant, anti-inflammatory, and antimicrobial effects [13,14,15,16]. Through mechanical and chemical processing, high-value-added extracts can be obtained, among which α-mangostin stands out as the principal xanthone. MPE exhibits potent free-radical scavenging capacity and modulates redox-sensitive signaling networks [12,17], and it has been shown to attenuate intestinal inflammation, suppress tumorigenesis, and improve metabolic balance with low toxicity and good bioavailability [18,19]. Although MEP and its major bioactive compound α-mangostin have been extensively studied in mammals for their antioxidant and anti-inflammatory effects, research in poultry—especially in laying hens—remains limited. Despite its promise as a functional feed additive in poultry [20], it remains unclear whether MEP can protect against oxidative stress or how it may modulate the gut–liver axis to confer systemic protection in avian species.

We previously established a diquat (DQ)-induced oxidative stress model in laying hens, which effectively simulates tissue injury through the gut–liver axis [21]. Due to its robust oxidative potential, DQ has been shown to trigger hepatic injury alongside gut microbiota imbalance and barrier dysfunction in various animal models [22,23,24]. The NRF2/HO-1 pathway is essential for hepatic antioxidant defense in laying hens, reducing ROS and mitigating oxidative damage under stress conditions [25,26]. Activation of this pathway preserves liver health and improves laying performance by strengthening the antioxidant capacity of hepatocytes under oxidative stress. In this study, we explored the protective effects of MPE against diquat-induced hepatic oxidative injury in laying hens, with a particular focus on the NRF2/HO-1 antioxidant pathway and the gut–liver–microbiota axis. Comprehensive analyses were conducted, including hepatic histopathology, oxidative stress biomarkers, intestinal permeability, and gut microbial profiling based on 16S rDNA sequencing. To further clarify the microbiota-mediated contribution of MPE, fecal microbiota transplantation (FMT) was performed using microbial donors from control and MPE-treated birds. This strategy enabled us to determine whether modulation of the gut microbiota plays a causal role in the hepatic protection conferred by MPE. These findings provide novel mechanistic insights into the microbiota-associated redox regulatory effects of MPE and support its application as a functional dietary intervention for mitigating oxidative stress and hepatic damage in poultry.

## 2. Materials and Methods

### 2.1. Ethics Statement

All experimental procedures followed institutional guidelines and were approved by the Animal Care and Use Committee of Zhejiang University (Hangzhou, China; Approval No. ZJU20241152).

### 2.2. Experimental Design and Diets

A total of 270 Hy-Line White laying hens, aged 40 weeks and exhibiting uniform body weight and egg-laying performance, were randomly allocated to three groups. Each group included six replicates, with 15 birds per replicate. The three groups were the control group (CON), the diquat-challenged group (DQ), and the MEP intervention with the diquat-challenged group (MQ). The fine powdered MPE was thoroughly premixed into the basal diet to ensure uniform distribution before feeding. After a 2-week adaptation period, hens in the CON and DQ groups were fed basal diets, while the hens in the MQ groups were fed diets with 50 mg/kg of MEP. The MEP was sourced from Zhejiang Boxin Pharmaceutical Co., Ltd. (Huzhou, China), of which more than 80% is α-mangostin. The experimental diets (Table S1) were formulated according to the NRC (1994) nutrient requirements. After 10 weeks of dietary intervention, birds in the DQ and MQ groups were intraperitoneally injected once with diquat (10 mg/kg body weight) to induce oxidative stress, as previously described [21], while the CON group received an equal volume of PBS. The DQ solution (200 g/L) was obtained from the Institute of Plant Protection, Chinese Academy of Agricultural Sciences (Beijing, China). All hens were housed in an environmentally controlled room (24 ± 3 °C, 50–60% relative humidity) with a 16 h light and 8 h dark photoperiod, and they had free access to feed and water. The birds were monitored daily, and samples were collected one week after DQ injection.

### 2.3. Sample Collection

At the end of the experiment, one hen per replicate (*n* = 6 per group) whose body weight was closest to the group mean was euthanized by cervical dislocation for sample collection. Blood was drawn from the wing vein and centrifuged at 3000× *g* for 10 min at 4 °C, and the resulting serum was immediately stored at −80 °C. The liver, abdominal fat, jejunum, and cecum were promptly excised. Liver samples were divided into portions for different analyses: one portion was fixed in 4% paraformaldehyde for histological examination, another portion was fixed in 2.5% glutaraldehyde for transmission electron microscopy to evaluate mitochondrial ultrastructure, and the remainder was snap-frozen in liquid nitrogen and stored at −80 °C for molecular and biochemical assays. Mid-jejunum tissues were likewise collected, with portions preserved in 4% paraformaldehyde for histological analysis and in 2.5% glutaraldehyde for transmission electron microscopy. Cecal contents were collected aseptically and rapidly frozen in liquid nitrogen for subsequent microbiota analysis.

### 2.4. Fecal Microbiota Transplantation

Thirty hens that had been previously subjected to DQ challenge to induce oxidative stress and hepatic injury, following previously established oxidative stress models in poultry, were randomly divided into two groups: FMT-CON and FMT-MG (*n* = 15 per group). All birds received a broad-spectrum antibiotic cocktail via oral gavage for seven consecutive days to deplete the endogenous gut microbiota. This was followed by daily oral administration of 5 mL/kg freshly prepared fecal suspension for three weeks. Fecal donors were selected from the CON and MQ groups. Fresh fecal samples were collected daily, excluding the white uric acid portion, suspended in sterile phosphate-buffered saline (PBS) at a ratio of 1 g feces to 5 mL PBS, homogenized, and centrifuged at 500× *g* for 1 min at 4 °C to remove large particles, and the resulting supernatant was filtered through sterile gauze to eliminate finer debris. The preparation of the antibiotic cocktail and fecal suspension was performed as previously described in our earlier study [27]. At the end of the experiment, one hen per replicate (*n* = 6 per group) whose body weight was closest to the group mean was euthanized by cervical dislocation for sample collection.

### 2.5. Organ Index

Following euthanasia, abdominal adipose and hepatic tissues were immediately excised and weighed. Organ indices were calculated by normalizing the tissue weight to the corresponding live body weight using the following formula: Organ index (g/kg) = organ weight (g)/body weight (kg).

### 2.6. Serum Biochemical Profiling

Serum biochemical parameters were assessed based on established protocols from our previous work [28]. Specifically, commercial assay kits were employed to quantify markers of liver function (ALT and AST) and lipid metabolism (TG, TC, HDL-C, and LDL-C) using an automatic biochemical analyzer (UA5800, Beckman Coulter, Drive Indianapolis, IN, USA) in accordance with the manufacturers’ guidelines.

### 2.7. Histological Analysis

Hepatic and jejunum tissues fixed in 4% paraformaldehyde were embedded in paraffin, sectioned at a thickness of 5 μm, and stained with hematoxylin and eosin (H&E), as Ge et al. [29] previously described. Stained slides were examined and photographed using a light microscope (Nikon Eclipse 80i, Nikon, Tokyo, Japan). Villus height (VH) and crypt depth (CD) in the jejunum were measured quantitatively with ImageJ software version 1.53 (NIH, Bethesda, MD, USA). For each group, six biological replicates were analyzed, with six measurements per replicate to ensure analytical reliability.

### 2.8. Oil Red O Staining and Sirius Red Staining

Hepatic tissues were embedded in optimal cutting temperature (OCT) compound and sectioned into frozen slices. The sections were stained with Oil Red O following standard protocols. Stained sections were observed and imaged using an optical microscope (Nikon Eclipse 80i, Nikon, Japan). For Sirius Red staining, paraffin-embedded liver sections were deparaffinized, rehydrated, and stained with Sirius Red solution. After staining, sections were rinsed, mounted, and examined under the same microscope.

### 2.9. Transmission Electron Microscopy (TEM)

Hepatic and jejunal tissues were cut into ~1 mm^3^ fragments, fixed in glutaraldehyde, and dehydrated through a graded ethanol series. The samples were then embedded, sectioned into ultrathin slices, and stained with uranyl acetate and lead citrate. Ultrastructural features were examined using a transmission electron microscope (JEOL JEM-1200EX, Peabody, MA, USA) following the protocol described by Liu et al. [30].

### 2.10. Intestinal Permeability Parameters

Serum levels of lipopolysaccharide (LPS), diamine oxidase (DAO), and D-(-)-lactic acid (D-LA) were measured using commercial assay kits (Aifang, Changsha, China) following the manufacturer’s instructions.

### 2.11. Oxidative Stress Parameter Assays

Liver tissues and serum were analyzed for total antioxidant capacity (T-AOC), catalase (CAT), glutathione peroxidase (GSH-PX), superoxide dismutase (SOD), and malondialdehyde (MDA) levels using commercial assay kits (Jiancheng Bioengineering Institute, Nanjing, China). Results were standardized based on total protein concentration for cross-sample consistency.

### 2.12. Immunofluorescence Analysis

Paraffin-embedded jejunum and liver tissue sections were deparaffinized, subjected to antigen retrieval, and treated with a fluorescence quenching reagent, followed by blocking with normal serum. Sections were then incubated overnight at 4 °C with primary antibodies against Occludin-1, MUC-2, NRF-2, and HO-1. After thorough washing, fluorophore-conjugated secondary antibodies matched to the host species were applied. Cell nuclei were stained with DAPI, and tissue sections were sealed using an anti-fade mounting reagent. Fluorescence signals were detected and captured using a fluorescence microscope, following previously reported procedures [27].

### 2.13. Enzyme-Linked Immunosorbent Assay (ELISA)

Serum concentrations of inflammatory cytokines (IL-6, IL-10, TNF-α, and TGF-β) were quantified in liver homogenate supernatants using chicken-specific ELISA kits (ABclonal Technology, Wuhan, China), as described previously [4]. To account for variations in tissue composition, all cytokine levels were normalized relative to the total protein content.

### 2.14. RNA Extraction and Quantitative Real-Time PCR Analysis

Total RNA was extracted from homogenized liver tissues and jejunum tissues using FreeZol™ Reagent (R711-01, Vazyme Biotech, Nanjing, China) and subsequently reverse-transcribed into complementary DNA (cDNA) using the HiScript^®^ III First Strand cDNA Synthesis Kit (R312-01, Vazyme) following the manufacturer’s protocol. Quantitative real-time PCR (qRT-PCR) was performed on a Bio-Rad CFX Connect™ Real-Time PCR System with Taq Pro Universal SYBR^®^ qPCR Master Mix (Q712-02, Vazyme). Each 20 µL reaction contained 10 µL of SYBR Master Mix, 1 µL of cDNA, 0.4 µL of forward and reverse primers (10 µM), and 8.2 µL of nuclease-free water. The amplification protocol was as follows: initial denaturation at 95 °C for 30 s, followed by 40 cycles of denaturation at 95 °C for 5 s, annealing at 60 °C for 30 s, and extension at 72 °C for 30 s. Specifically, all primers were designed using NCBI Primer-BLAST based on Gallus gallus mRNA sequences retrieved from the GenBank database, ensuring high specificity and amplification efficiency. The primer sequences are listed in Table S2. β-actin was selected as the reference gene after confirming its stable expression across all experimental groups and tissues using normalization analysis, ensuring its suitability for accurate gene expression quantification. Relative mRNA expression levels were normalized to β-actin and quantified using the 2^−ΔΔCt^ method [31].

### 2.15. Western Blotting

Total protein was extracted from liver tissues and separated by 12% SDS-PAGE (GenScript, Nanjing, China), followed by transfer onto nitrocellulose membranes (Millipore, MA, USA). Membranes were blocked with 5% skim milk and incubated overnight at 4 °C with primary antibodies against β-ACTIN (EM21002), NRF2 (R1312-8), and HO-1 (HA721854) (HUABIO, Hangzhou, China) under gentle agitation. After washing, the membranes were incubated at 37 °C for 1 h with an HRP-conjugated universal secondary antibody (goat anti-rabbit/mouse, HUABIO). Protein bands were visualized using the Tanon 5200 detection system (Tanon, Shanghai, China), and band intensities were quantified using ImageJ software (NIH, USA), with β-ACTIN serving as the internal loading control.

### 2.16. 16S rDNA Sequencing Analysis

Following total genomic DNA extraction and quality assessment, the V3-V4 region of the 16S rDNA gene was amplified with the 338F and 806R primer pair and sequenced using the Illumina MiSeq platform. Sequences were clustered into operational taxonomic units (OTUs) via the UPARSE algorithm (v7.0.1001). Beta diversity analysis and linear discriminant analysis (LDA) were conducted using QIIME software (version 1.9.1) and LEfSe method, respectively. Relative abundance and statistical comparisons of microbiota at the phylum, family, and genus levels, along with correlation analysis and heatmap visualization, were performed using R software (version 4.4.2).

### 2.17. Statistical Analysis

All data were tested for normal distribution using the Shapiro–Wilk test before performing one-way ANOVA. Statistical analyses were performed using one-way ANOVA in IBM SPSS Statistics version 26.0, followed by Tukey’s post hoc test for multiple comparisons. A *p*-value < 0.05 was considered statistically significant. Data are presented as means ± standard deviations (SDs). Graphs were generated using GraphPad Prism version 9.5.

## 3. Results

### 3.1. MPE Ameliorated DQ-Induced Hepatic Oxidative Injury

**Figure 1:**
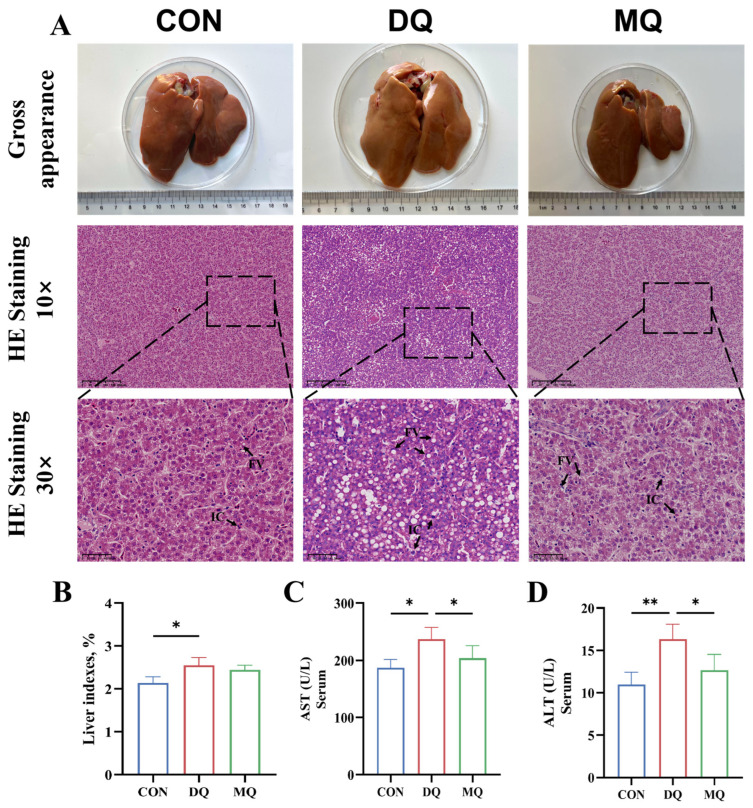
Mangosteen pericarp extracts ameliorated DQ-induced hepatic injury. (**A**) Gross appearance of liver morphology and representative image of H&E staining of liver morphology. (**B**–**D**) Liver index and ALT and AST activity in serum. Representative images of H&E staining (10×, scale bar = 200 μm; 30×, scale bar = 50 μm). Abbreviations: AST, aspartate aminotransferase; ALT, alanine transaminase. All data are represented as mean ± SD. *n* = 6 hens per group. * *p* < 0.05, ** *p* < 0.01.

As shown in Figure 1A, the livers from the DQ group appeared yellowish with visible hemorrhagic spots, while the control livers had a smooth, reddish-brown appearance. In contrast, the MQ group displayed more normal liver coloration and morphology, indicating that MPE supplementation alleviated DQ-induced gross hepatic alterations. H&E staining revealed extensive lipid vacuole accumulation and increased infiltration of inflammatory cells in DQ livers, whereas the MQ group showed reduced lipid droplet density and markedly less inflammatory infiltration, approaching the histological profile of the control group. Additionally, the liver index was significantly higher in the DQ group compared to the CON group (*p* < 0.05), while the MQ group showed a moderate reduction, though the difference was not statistically significant (Figure 1B). Serum levels of ALT and AST were significantly elevated in DQ-treated hens compared to the CON group (*p* < 0.05), and MPE treatment significantly reduced both markers (*p* < 0.05; Figure 1C,D), indicating its hepatoprotective efficacy.

### 3.2. MPE Alleviated DQ-Induced Hepatic Fibrosis and Lipid Deposition

**Figure 2:**
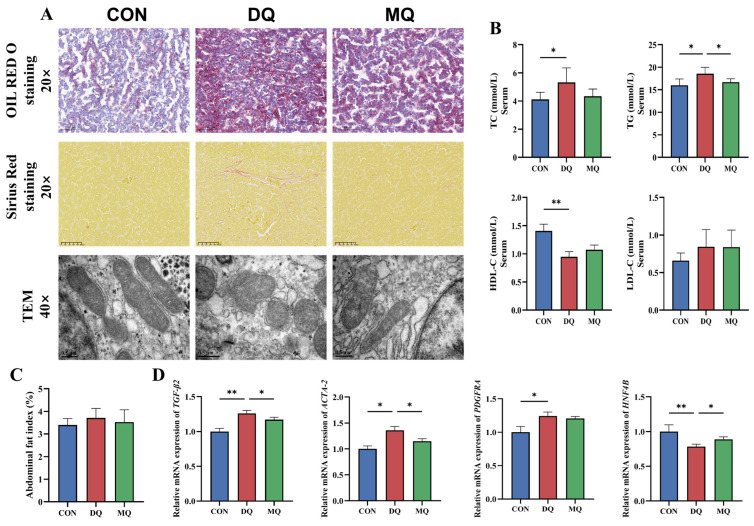
Mangosteen pericarp extracts alleviated DQ-induced hepatic fibrosis and lipid disorders. (**A**) Representative images of Oil Red O staining and Sirius Red staining, and representative TEM images of liver mitochondria. (**B**) Serum lipid metabolism-related indexes of laying hens. (**C**) Weight of abdominal adipose as percentage of body weight. (**D**) mRNA expression of relevant genes reflecting degree of liver fibrosis. Representative images of Oil Red O staining (20×, scale bar = 100 µm), Sirius Red staining (20×, scale bar = 100 µm), and TEM (40×, scale bar = 500 nm). Abbreviations: TC, total cholesterol; TG, triglyceride; HDL-C, high-density lipoprotein cholesterol; LDL-C, low-density lipoprotein cholesterol; TGF-β2, transforming growth factor beta-2; ACTA-2, actin αlpha-2; PDGFRA, platelet-derived growth factor receptor alpha; HNF4B, hepatocyte nuclear factor 4 beta; all data are represented as mean ± SD. *n* = 6 hens per group. * *p* < 0.05, ** *p* < 0.01.

Figure 2A shows that Oil Red O staining highlighted considerable lipid accumulation in the DQ group compared to the CON group, whereas MPE treatment mitigated hepatic steatosis. Sirius Red staining indicated increased collagen deposition, reflecting enhanced hepatic fibrosis following DQ exposure, which was markedly reduced by MPE. TEM further showed that DQ caused mitochondrial loss, swelling, vacuolization, crista disruption, and lipid droplet accumulation. These ultrastructural damages were notably improved in the MQ group, with better-preserved mitochondria and fewer lipid droplets. Biochemical analysis demonstrated that DQ significantly increased TC and TG levels and decreased HDL-C (*p* < 0.05; Figure 2B), whereas MPE reversed these changes and significantly lowered TG levels. At the transcriptional level, DQ upregulated *TGF-β2*, *ACTA-2*, and *PDGFRA* and downregulated *HNF4B* expression compared to controls (*p* < 0.05; Figure 2D). MQ treatment restored these gene expressions, indicating mitigation of liver fibrosis.

### 3.3. MPE Enhanced Antioxidant Capacity in DQ-Induced Oxidative Stress

**Figure 3:**
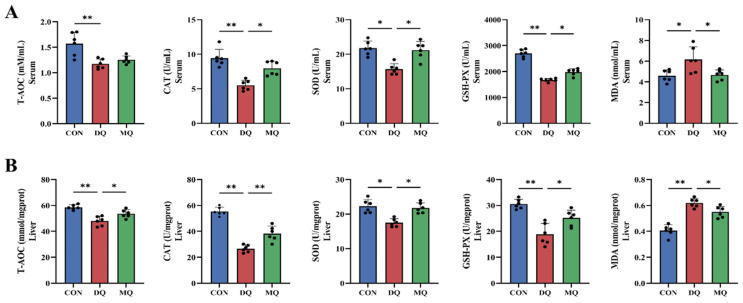
Mangosteen pericarp extracts enhanced antioxidant capacity in DQ-induced laying hens. (**A**,**B**) Levels of hepatic oxidative stress-related parameters T-AOC, CAT, GSH-PX, SOD, and MDA in serum and liver. Abbreviations: T-AOC, total antioxidant capacity; CAT, catalase; SOD, superoxide dismutase; GSH-Px, glutathione peroxidase; MDA, malondialdehyde. All data are represented as mean ± SD. *n* = 6 hens per group. * *p* < 0.05, ** *p* < 0.01.

As shown in Figure 3A,B, DQ exposure significantly reduced the activities of T-AOC, GSH-Px, and SOD in both serum and liver (*p* < 0.05), while MDA levels were significantly elevated. In contrast, MPE supplementation significantly increased the activities of these antioxidant enzymes and reduced MDA content compared to the DQ group. Further analysis revealed that dietary MPE increased the activities of CAT, GSH-Px, and SOD in serum, and T-AOC, CAT, SOD, and GSH-Px in liver tissue, while significantly reducing MDA levels in both serum and liver (*p* < 0.05). These findings indicate that MPE supplementation enhanced antioxidant enzyme activity and mitigated the oxidative effects induced by DQ.

### 3.4. MPE Attenuated DQ-Induced Hepatic Oxidative Stress by Activating the NRF2/HO-1 Pathway

**Figure 4:**
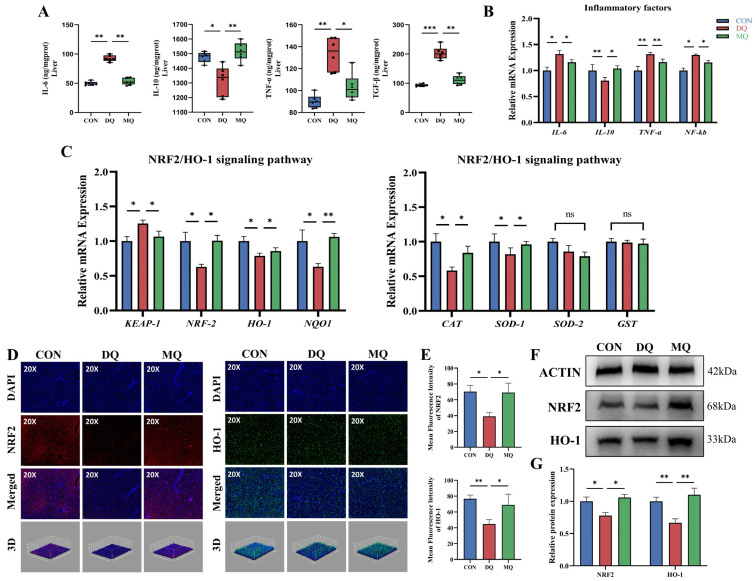
Mangosteen pericarp extracts attenuated DQ-induced hepatic oxidative stress by activating the NRF2/HO-1 pathway. (**A**) The levels of hepatic inflammation-related parameters IL-6, IL-10, TNF-α, and TGF-β. (**B**) The relative mRNA expression of genes related to the inflammatory response. (**C**) The relative mRNA expression of the downstream target gene. (**D**,**E**) The representative hepatic NRF2 and HO-1 fluorescence staining images and quantitative results. (**F**,**G**) The representative western blot images and relative expression levels of NRF2 and HO-1 protein in the CON, DQ, and MQ groups. Representative images of fluorescence staining (20×, scale bar = 50 μm). Abbreviations: TNF-α, TNF-alpha; IL-6, interleukin 6; IL-10, interleukin 10; NF-κB, nuclear factor kappa B; NRF2, nuclear factor erythroid 2-related factor 2; HO-1, heme oxygenase-1; KEAP-1, Kelch-like ECH-associated protein-1; NQO1, NAD(P)H: quinone oxidoreductase-1; CAT, catalase; SOD-1, superoxide dismutase-1; SOD-2, superoxide dismutase-2; GST, glutathione S-transferase. All data are represented as the mean ± SD. *n* = 6 hens per group. * *p* < 0.05, ** *p* < 0.01, *** *p* < 0.001, and ns indicates an insignificant difference (*p* > 0.05).

To explore the molecular mechanisms behind the hepatoprotective effects of α-mangostin, we assessed the activation of the NRF2/HO-1 signaling pathway in DQ-induced hepatic oxidative stress. As shown in Figure 4A, serum levels of pro-inflammatory cytokines IL-6 and TNF-α were significantly higher in the DQ group than in the CON group (*p* < 0.01), while the anti-inflammatory cytokine IL-10 was notably decreased (*p* < 0.05), and the fibrogenic marker TGF-β was significantly elevated (*p* < 0.01). These alterations were significantly reversed by MPE treatment, suggesting its anti-inflammatory and anti-fibrotic effects. Consistently, RT-qPCR analysis revealed significant dysregulation of hepatic mRNA levels of *IL-6*, *TNF-α*, *IL-10*, and *NF-κB* in the DQ group, with *IL-10* significantly downregulated (*p* < 0.05). MPE supplementation effectively restored these inflammatory markers toward control levels (Figure 4B). To assess the involvement of NRF2 signaling, we analyzed the expression of key genes within the pathway. As shown in Figure 4C, DQ exposure significantly downregulated the expression of *NRF2*, *HO-1*, and *NQO1*, and it upregulated *KEAP1*, a negative regulator of *NRF2* (*p* < 0.05). MPE treatment significantly reversed these alterations. The expression levels of antioxidant enzymes *CAT* and *SOD1* were moderately improved by MPE, while *SOD2* and *GST*, although suppressed by DQ, remained unchanged after treatment. Immunofluorescence analysis (Figure 4D,E) further confirmed that DQ reduced hepatic expression of NRF2 and HO-1, whereas MPE treatment significantly enhanced their fluorescence intensities (*p* < 0.05). These findings were corroborated by 3D fluorescence quantification. Western blotting results (Figure 4F,G) were consistent with RT-qPCR and fluorescence data, showing that DQ markedly reduced NRF2 and HO-1 protein levels, which were significantly restored by MPE supplementation (*p* < 0.05). Together, these findings suggest that α-mangostin alleviates DQ-induced hepatic oxidative stress by activating the NRF2/HO-1 signaling pathway, leading to reduced inflammation and enhanced antioxidant enzyme expression.

### 3.5. MPE Ameliorated DQ-Induced Intestinal Barrier Dysfunction and Mucosal Oxidative Injury

**Figure 5:**
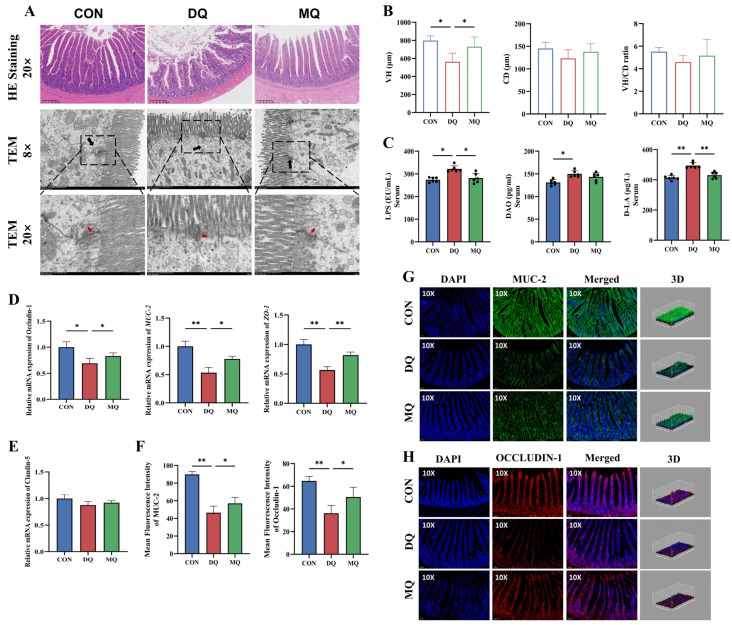
Mangosteen pericarp extracts ameliorated intestinal barrier dysfunction and mucosal injury under DQ-induced oxidative stress. (**A**) Representative images of H&E staining and TEM. (**B**) Villus height, crypt depth, and villus height to crypt depth ratio. (**C**) The intestinal permeability indicators LPS, DAO, and D-LA levels. (**D**,**E**) Relative mRNA expression levels of tight junction protein genes ZO-1, Occludin-1, Claudin-5, and mucin gene MUC-2. (**F**–**H**) Representative jejunal MUC-2 and Occludin-1 fluorescence staining images and quantitative results. Black and red arrows indicate tight junctions in the jejunum. Representative images of H&E staining (20×, scale bar = 100 μm), TEM (8×, scale bar = 2 μm), TEM (20×, scale bar = 500 nm) and fluorescence staining (10×, scale bar = 50 μm). Abbreviations: ZO-1, zonula occludens-1; MUC-2, Mucin 2. All data are represented as mean ± SD. *n* = 6 hens per group. * *p* < 0.05, ** *p* < 0.01.

As shown in Figure 5, MPE alleviated DQ-induced intestinal barrier disruption. H&E staining revealed intact villus structures in the CON group, while the DQ group showed notable mucosal injury and villus atrophy. MPE treatment significantly improved these histological alterations, with a notable increase in villus height (VH) compared to the DQ group (*p* < 0.05; Figure 5A,B). TEM analysis further showed that tight junctions were severely disrupted or even absent in the DQ group, whereas MPE supplementation restored tight junction integrity (Figure 5A). Consistent with structural damage, intestinal permeability was significantly increased after DQ exposure, as reflected by higher serum levels of LPS, DAO, and D-LA. Notably, MPE treatment significantly reduced LPS and D-LA levels compared to the DQ group (*p* < 0.05; Figure 5C). At the molecular level, MPE significantly upregulated mRNA expression of *MUC-2*, Occludin-1, and *ZO-1* (*p* < 0.05; Figure 5D). Further confirmation by immunofluorescence analysis confirmed the increased protein expression of MUC-2 and Occludin-1 in the MQ group (*p* < 0.05), indicating enhanced intestinal barrier repair (Figure 5F–H).

### 3.6. MPE Attenuated DQ-Induced Gut Microbiota Disorder

**Figure 6:**
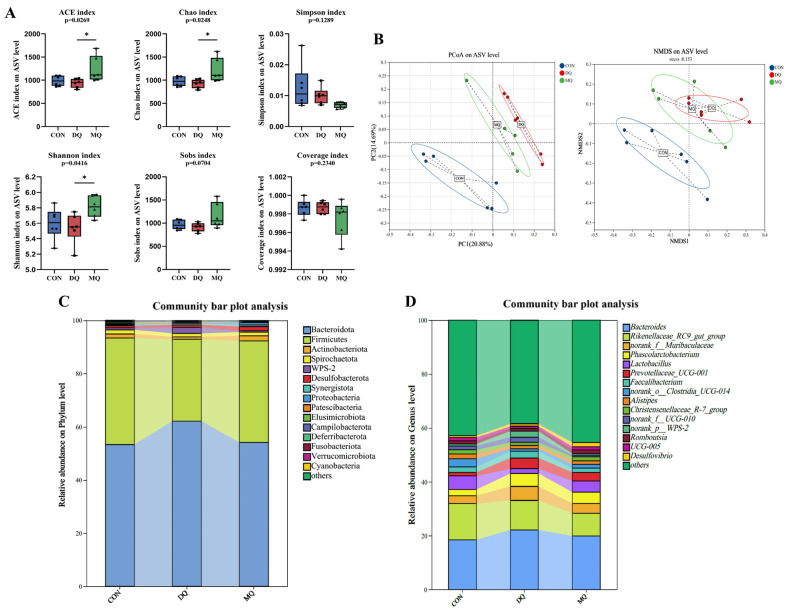
Mangosteen pericarp extracts altered the composition of the cecal microbiota. (**A**) The panels represent the Shannon index, Chao index, Simpson index, Sobs index coverage index, and ACE species of cecal microflora. (**B**) The principal coordinate analysis (PCoA) and non-metric multidimensional scaling (NMDS) score plot on the ASV level between the CON, DQ, and MQ groups. (**C**,**D**) The percent of community abundance of the cecal microflora community at the phylum and genus levels. The data are shown as the mean ± SD. *n* = 6 hens per group. * *p* < 0.05.

**Figure 7:**
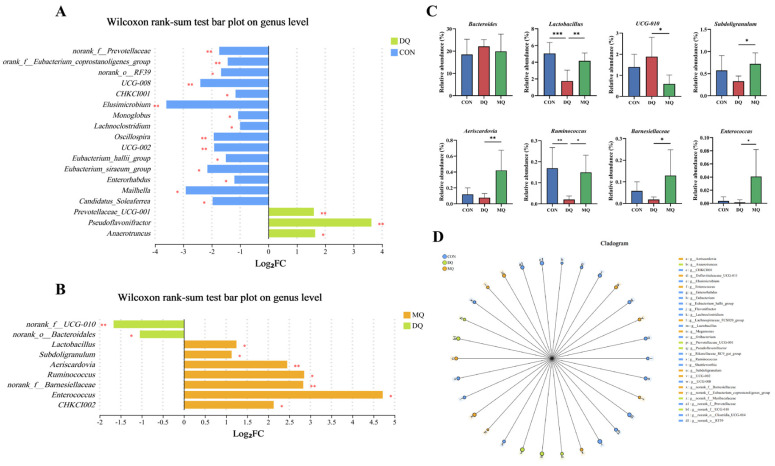
The changes in the cecal microbiota composition. (**A**) Wilcoxon rank-sum test bar plot on the genus level between the CON and DQ groups. (**B**) Wilcoxon rank-sum test bar plot on the genus level between the MQ and DQ groups. (**C**) The relative abundance of cecal microbiota at the genus level with significant variations and specific functions. (**D**) The cladogram of LEfSe analysis. The data are shown as the mean ± SD. *n* = 6 hens per group. * *p* < 0.05, ** *p* < 0.01, *** *p* < 0.001.

The α-diversity analysis showed that DQ exposure significantly reduced microbial richness and diversity, as indicated by lower ACE, Chao, and Shannon indices compared to the control group. MPE treatment restored these metrics, with the MQ group showing significant increases in ACE (*p* = 0.0269), Chao (*p* = 0.0248), and Shannon (*p* = 0.0416). The Simpson index was not statistically different, yet its downward trend in the MQ group corroborated the overall improvement in diversity; Sobs also displayed an upward tendency (*p* = 0.0704). Coverage values remained > 0.994 across all groups, confirming adequate sequencing depth (Figure 6A). Principal coordinate analysis (PCoA) and non-metric multidimensional scaling (NMDS) (Figure 6B) demonstrated clear separation among the CON, DQ, and MQ groups (PERMANOVA, *p* < 0.05), with PC1 and PC2 explaining 20.88% and 14.69% of the variance, respectively. Taxonomic profiling (Figure 6C,D) highlighted pronounced shifts at both the phylum and genus levels. To further explore the modulatory effects of MPE on gut microbial composition, we analyzed genus-level differences using Wilcoxon rank-sum tests. The Wilcoxon rank-sum tests showed a marked enrichment of the *UCG-010*, *Pseudoflavoniftactor*, and *Anaerotruncus* in the DQ group and significant losses of *norank_f__Prevotellaceae*, *CHKC1001*, *Oscillospira*, and *Lachnoclostridium* (Figure 7A,B). MPE supplementation reversed these alterations: *UCG-010* abundance declined significantly, whereas beneficial genera, including *Lactobacillus*, *Ruminococcus*, and *Subdoligranulum*, were enriched. Genus-level bar plots (*p* < 0.05; Figure 7C) mirrored this restoration, and LEfSe analysis (Figure 7D) confirmed distinct microbial signatures among groups. Collectively, these findings indicate that α-mangostin mitigates DQ-induced gut dysbiosis by suppressing potentially harmful bacteria and restoring beneficial commensals, thereby contributing to intestinal homeostasis and alleviating oxidative stress.

### 3.7. Correlation Analysis

**Figure 8:**
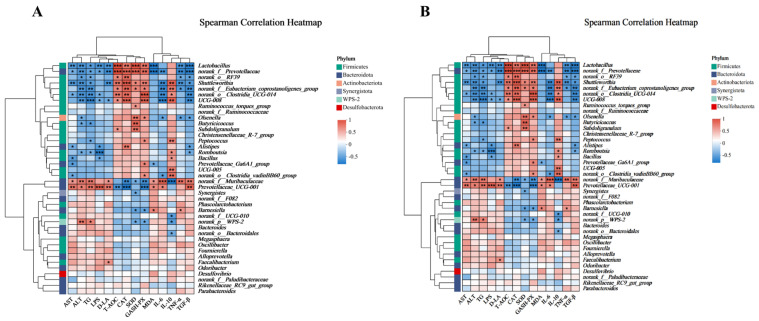
Correlation analysis between gut microbiota and oxidative stress and inflammatory parameters. (**A**) Heatmap of correlation analysis of cecal microbiota with indicators of liver injury and parameters related to oxidative stress and inflammation. (**B**) Heatmap of correlation analysis of cecal microbiota with representative genes associated with oxidative stress, tight junction, and anti-inflammatory cytokine. * *p* < 0.05, ** *p* < 0.01, *** *p* < 0.001.

Spearman correlation analysis was performed, and the heatmaps are displayed. As shown in Figure 8A, the anti-inflammatory cytokine IL-10 and the antioxidative factors (T-AOC, CAT, SOD, and GSH-PX) were similar in the correlation analysis clustering. Meanwhile, MDA, the pro-inflammatory factors (LPS, D-LA, IL-6, TNF-α, and TGF-β), and the markers of liver injury (AST, ALT, and TG) had similar patterns in the correlation analysis clustering. As shown in Figure 8B, the anti-oxidative-stress-related genes (*NRF-2*, *HO-1*, *NQO-1*, *CAT,* and *SOD-1*), the tight junction genes (*ZO-1*, *MUC-2*, and *Occludin-1*), and the anti-inflammatory cytokine (*IL-10*) were similar in the correlation analysis clustering. Correlation analysis clustering revealed similar patterns for the oxidative stress-related gene (*KEAP-1*) and inflammatory genes (*IL-6*, *TNF-α*, and *NF-κB*). The levels of anti-inflammatory cytokines and antioxidant factors were positively correlated with the relative abundance of *Lactobacillus*, *norank_f__Prevotellaceae*, *norank_o__RF39*, *Shuttleworthia*, *norank_f__Eubacterium_coprostanoligenes_group*, *norank_o__Clostridia_UCG-014,* and *UCG-008*, while they were negatively correlated with the abundance of *norank_f__Muribaculaceae* and *Prevotellaceae_UCG-001* (*p* < 0.05). The genes related to anti-oxidative stress, as well as the genes related to tight junction, were positively correlated with the relative abundance of *Lactobacillus*, *norank_f__Prevotellaceae*, *norank_o__Clostridia_UCG-014*, *UCG-008,* and *Shuttleworthia* and negatively correlated with the relative abundance of *norank_f__Muribaculaceae* and *Prevotellaceae_UCG-001* (*p* < 0.05).

### 3.8. FMT Further Verified MPE Attenuated Oxidative Stress Mediated by Gut Microbiota

**Figure 9:**
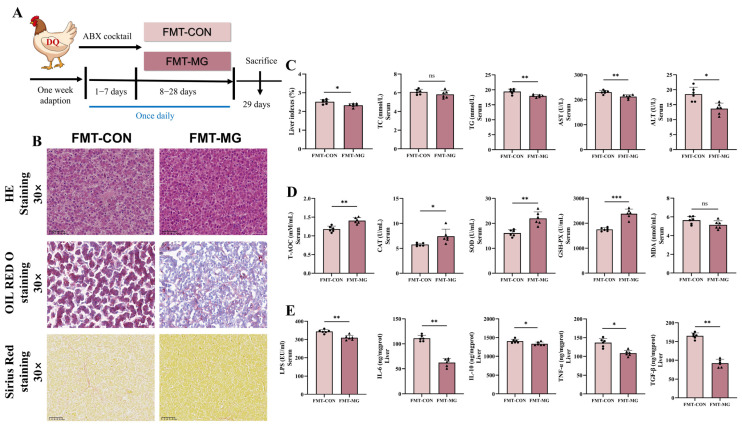
Fecal microbiota transplantation further highlights the role of gut microbiota modulation in mediating MPE’s systemic protective actions. (**A**) Schematic diagram of the experimental procedure. (**B**) Representative photomicrographs of liver tissues with H&E staining, Oil Red O staining, and Sirius Red staining images. (**C**) Liver indexes and serum biochemical indexes of laying hens. (**D**) The levels of hepatic oxidative stress-related parameters of serum T-AOC, CAT, GSH-PX, SOD, and MDA. (**E**) The intestinal permeability indicators LPS levels of serum, the levels of hepatic inflammation-related parameters IL-6, IL-10, TNF-α, and TGF-β. Representative images of H&E staining (30×, scale bar = 50 μm), Oil Red O staining (30×, scale bar = 50 μm), and Sirius Red staining (30×, scale bar = 50 μm). All data are represented as the mean ± SD. *n* = 6 hens per group. * *p* < 0.05, ** *p* < 0.01, *** *p* < 0.001.

To explore the therapeutic effects of gut microbiota modulation on oxidative stress, an FMT experiment was performed. In this study, microbiota from MPE-treated hens were transplanted into recipient hens that had been subjected to DQ-induced oxidative stress (Figure 9A). H&E and Oil Red staining revealed that the FMT-MG group had fewer lipid droplets and fat vacuoles than the FMT-CON group. Sirius red staining showed a significant reduction in collagen deposition, indicating that MPE-treated hens helped alleviate hepatic cellular infiltration and fibrosis (Figure 9B). Relative to the FMT-CON group, the FMT-MG group exhibited a significant decrease in liver index (*p* < 0.05; Figure 9C), suggesting a protective effect on liver morphology. Quantitative analysis of serum biomarkers revealed that the FMT-MG group exhibited significantly lower levels of liver injury markers, such as ALT and AST (Figure 9C), and improved lipid regulation, as evidenced by reduced TG levels. The FMT-MG group also exhibited significant improvements in antioxidant enzyme activity. The serum levels of T-AOC, SOD, and GSH-Px were significantly increased, while MDA levels were notably reduced (*p* < 0.05; Figure 9D), reflecting an enhanced systemic antioxidant defense and alleviation of oxidative damage caused by DQ exposure. Furthermore, inflammatory markers were significantly reduced in the FMT-MG group. Serum levels of LPS, IL-6, TNF-α, and TGF-β were significantly decreased in the FMT-MG group compared to the FMT-CON group (*p* < 0.05; Figure 9E). The reduction in LPS levels suggests that FMT from MPE-treated hens restores intestinal permeability, thereby reducing systemic inflammation. These findings provide further evidence that the MPE-mediated alleviation of oxidative stress is influenced by alterations in the gut microbial community.

## 4. Discussion

Oxidative stress has become a key limiting factor in modern poultry production, with significant implications for the poultry industry. Diquat (DQ), a widely used bipyridyl herbicide, effectively controls weeds but poses considerable environmental and biological risks due to its persistence and toxicity [32]. Given its strong pro-oxidant properties, DQ has been increasingly employed as a chemical inducer of oxidative stress in experimental models [33,34,35]. In the present study, we successfully established a DQ-induced oxidative stress model in laying hens to investigate hepatic and intestinal responses. Our results demonstrated that DQ exposure caused a marked redox imbalance, as indicated by reduced T-AOC and decreased activities of CAT, GSH-Px, and SOD, along with elevated MDA levels. These observations align with previous findings in other animal models [21,36,37]. Concurrently, DQ induced notable hepatic injury, lipid accumulation, inflammatory infiltration, and fibrosis, highlighting the extent of liver damage caused by oxidative stress. Additionally, we observed intestinal barrier dysfunction, including villus atrophy, tight junction disruption, and increased intestinal permeability. Together, these findings confirm that DQ induces severe oxidative stress and multi-organ damage, providing a robust platform to evaluate potential protective interventions.

Liver injury induced by DQ is primarily mediated through the excessive production of ROS, which induces oxidative stress, impairs antioxidant defenses, and ultimately leads to hepatocellular dysfunction [32]. Serum ALT and AST are well-established biomarkers of hepatic injury [38], and their significant elevation in the DQ-treated group in this study indicates substantial liver damage. Notably, MPE supplementation markedly reduced ALT and AST levels, suggesting its capacity to alleviate DQ-induced hepatic injury. In addition, DQ exposure disrupted lipid metabolism, as evidenced by increased serum levels of TC and TG, along with decreased HDL-C, which are metabolic disturbances commonly associated with hepatic dysfunction [39,40]. Treatment with MPE significantly lowered TG levels, reflecting its potential to restore lipid homeostasis and reduce hepatic lipid accumulation. Collectively, the reduction of ALT, AST, and TG levels in the MPE group underscores its protective role in maintaining liver function and metabolic balance under oxidative stress.

Liver fibrosis is a common pathological outcome of prolonged oxidative stress and chronic inflammation [41,42], characterized by hepatic stellate cell (HSC) activation and excessive extracellular matrix (ECM) deposition [43]. In this study, DQ exposure significantly increased the expression of fibrosis-related markers, including TGF-β2 and ACTA-2, indicating the initiation of fibrogenic processes. TGF-β2 is a key pro-fibrotic cytokine that promotes fibroblast activation and collagen synthesis [44,45], while ACTA-2 reflects the transformation of HSCs into myofibroblast-like cells that contribute to ECM accumulation [46,47]. Furthermore, DQ also elevated PDGFRA and pro-inflammatory cytokines such as IL-6 and TNF-α, which are known to interact with TGF-β and promote HSC activation and fibrosis progression [48,49]. These changes indicate that oxidative stress from DQ not only drives fibrotic gene expression but also enhances inflammation-related pathways that aggravate liver damage. MPE treatment significantly downregulated *TGF-β2*, *ACTA-2*, and *PDGFRA* expression, and it reduced IL-6 and TNF-α levels, suggesting that it can inhibit both fibrogenesis and inflammatory responses. Sirius Red staining confirmed decreased collagen accumulation, and TEM analysis revealed improved mitochondrial integrity, supporting its protective effect. Overall, MPE effectively mitigated DQ-induced liver fibrosis, likely by reducing oxidative stress and inflammation. This aligns with previous studies showing that MPE can modulate fibrosis-related gene expression and slow liver fibrosis progression [50,51].

This study demonstrated that DQ exposure induced severe oxidative stress in laying hens, as evidenced by reduced hepatic and systemic antioxidant enzyme activities (T-AOC, SOD, CAT, and GSH-Px) and increased MDA levels, similar to findings by Zhou et al. [52] and Zhan et al. [21]. These alterations reflect impaired antioxidant defense. Notably, MPE supplementation significantly restored antioxidant enzyme activity and reduced MDA levels, suggesting a potential antioxidative effect. Among these enzymes, SOD and GSH-Px are key components in the enzymatic detoxification of ROS, with SOD catalyzing the dismutation of superoxide radicals and GSH-Px converting hydrogen peroxide into water [53,54], thereby protecting cellular components from oxidative injury. The recovery of their activities in the MPE-treated group suggests a reactivation of endogenous antioxidant defense mechanisms. Moreover, the observed increase in T-AOC—a comprehensive indicator of systemic antioxidant capacity—suggests that MPE supplementation may contribute to enhancing the host’s redox defense mechanisms under DQ-induced stress [55]. This biochemical improvement was accompanied by morphological evidence of improved hepatic ultrastructure, including more preserved mitochondria and reduced lipid droplet accumulation, indicating that MPE may exert antioxidant effects at both cytoplasmic and subcellular levels. Given the liver’s central role in oxidative metabolism and its known sensitivity to ROS-induced damage [56], these findings support the notion that MPE can alleviate hepatic oxidative injury in this poultry model.

The NRF2/HO-1 signaling pathway is a critical regulator of the body’s antioxidant response, particularly in the liver, where it helps maintain redox balance and protect against oxidative damage [57,58]. Under basal conditions, NRF2 is retained in the cytoplasm by KEAP1, which targets it for proteasomal degradation. Upon oxidative stress, modifications to KEAP1 cysteine residues disrupt the NRF2-KEAP1 complex, enabling NRF2 to translocate to the nucleus, bind antioxidant response elements (AREs) in DNA [59], and activate cytoprotective genes, including HO-1 [60]. HO-1 then catalyzes heme degradation into biliverdin, free iron, and carbon monoxide, molecules that themselves exert antioxidant, anti-inflammatory, and anti-apoptotic effects [61]. In the present study, DQ exposure significantly inhibited NRF2 nuclear accumulation and HO-1 expression, indicating that DQ-induced oxidative stress not only elevated ROS production but also disrupted the very system designed to quench it. Concomitant upregulation of KEAP1 further confirmed the impaired NRF2 activation and a failure to mount an effective antioxidant defense [23,62]. Remarkably, MPE treatment reversed these effects by reducing KEAP1 mRNA levels, restoring NRF2 nuclear translocation, and significantly increasing HO-1 transcription and protein abundance. Several phytochemicals and natural feed additives, such as curcumin, resveratrol, and green tea polyphenols, have been extensively studied for their ability to alleviate oxidative stress in poultry through activation of the NRF2 pathway, suppression of inflammation, and improvement of gut health [63,64,65]. Similar to these agents, MPE supplementation reactivated the NRF2/HO-1 axis, leading to increased T-AOC, SOD, and GSH-Px activities and reduced MDA accumulation. This demonstrates that MPE not only alleviates oxidative stress but also strengthens the enzymatic defense against ROS. These findings emphasized the potential role of MPE in regulating redox in liver health.

The intestinal barrier is essential for protecting the body from harmful substances and maintaining gut homeostasis [66]. DQ-induced oxidative stress disrupts intestinal morphology and tight junction integrity, resulting in increased intestinal permeability and barrier dysfunction [67]. In line with this, our study showed that DQ exposure caused villus atrophy, tight junction disruption, and elevated intestinal permeability. Such damage impairs nutrient absorption and facilitates the translocation of toxins and pathogens, aggravating systemic inflammation and oxidative injury. Notably, MPE treatment significantly mitigated these effects, restoring barrier integrity. H&E staining and TEM analysis demonstrated preservation of villus structure and tight junction continuity, while MPE markedly upregulated the expression of the key tight junction proteins Occludin-1, ZO-1, and MUC-2, which are critical for regulating paracellular permeability, maintaining mucus layer stability, and supporting barrier function. MUC-2 is the predominant secreted mucin forming the protective mucus layer, while Occludin-1 and ZO-1 are integral to tight junction complexes, working in concert with claudins to seal the paracellular space and maintain intestinal integrity [68,69]. This recovery highlights MPE’s ability to repair and stabilize the intestinal barrier under oxidative stress. Moreover, improved barrier function was reflected by the reduced serum levels of LPS and D-LA, established markers of increased gut permeability and barrier dysfunction. Elevated LPS and D-LA promote systemic inflammation and oxidative stress [70], but their decrease after MPE treatment indicates effective prevention of endotoxin translocation. Together, these results suggest that MPE protects the intestinal barrier, thereby mitigating DQ-induced systemic oxidative damage.

Disruption of gut microbial homeostasis is increasingly recognized as a key contributor to oxidative stress and liver injury via the gut–liver axis [71,72]. In this study, DQ exposure led to a reduction in microbial diversity, as evidenced by decreased ACE, Chao, and Shannon indices, along with notable taxonomic shifts—characterized by a decline in beneficial genera, such as *Lactobacillus*, *Ruminococcus*, and *Subdoligranulum* [73,74], and the enrichment of potentially pathogenic taxa, including *Bacteroidales*, *UCG-010*, *Pseudoflavonifractor*, and *Anaerotruncus* [75,76,77]. These changes are indicative of a dysbiotic state that may aggravate oxidative damage and inflammation. Remarkably, MPE supplementation reversed these microbiota alterations, restoring microbial diversity and promoting the enrichment of commensals associated with anti-inflammatory and antioxidant effects, such as *Lactobacillus*, *Subdoligranulum*, *Ruminococcus*, and *Aeriscardovia*. Further supporting this, Spearman correlation analysis revealed that beneficial genera such as *Lactobacillus*, *Clostridia_UCG-014*, and *Shuttleworthia* were strongly associated with antioxidant parameters (T-AOC, SOD, GSH-Px), tight junction gene expression (*ZO-1*, *Occludin-1*), and IL-10, while they were negatively associated with pro-inflammatory and oxidative markers. These correlations suggest a potential role for microbiota modulation in mediating the systemic protective effects of MPE [78]. In summary, MPE not only counteracts hepatic oxidative stress through direct antioxidant pathways but also improves gut microbial composition, which may synergistically contribute to intestinal and hepatic protection under oxidative challenge.

To further verify the causal role of gut microbiota in mediating the protective effects of MPE, we performed an FMT experiment. Transplantation of microbiota from MPE-treated donors into DQ-exposed recipients significantly alleviated hepatic injury, as evidenced by improved liver morphology, reduced lipid accumulation and fibrosis, and restoration of antioxidant enzyme activities. Simultaneously, pro-inflammatory markers like LPS, TNF-α, and IL-6 were significantly reduced, and hepatic oxidative stress was alleviated. These results support the idea that gut microbiota modulation is a key mechanism through which MPE exerts its hepatoprotective effects.

Several phytochemicals and natural feed additives, such as curcumin, resveratrol, and green tea polyphenols, have been extensively studied for their ability to alleviate oxidative stress in poultry. These compounds typically exert their effects through the activation of the NRF2 pathway, suppression of inflammation, and improvement of gut health [63,64,65]. Similar to these agents, MPE effectively restored redox balance and reduced hepatic injury; however, MPE is distinguished by its high α-mangostin content and its combined actions on hepatic antioxidant defense, intestinal barrier integrity, and gut microbiota modulation. This multifaceted mode of action suggests that MPE may offer broader protective benefits under oxidative stress conditions, positioning it as a promising candidate for integration into poultry feeding strategies aimed at enhancing resilience, productivity, and overall health.

## 5. Conclusions

This study demonstrated that MPE can be an outstanding supplement for the prevention and mitigation of hepatic oxidative damage in laying hens. Mechanistically, MPE reactivated the NRF2/HO-1 antioxidant pathway and restored intestinal barrier integrity by upregulating tight junction protein expression (Occludin-1 and ZO-1) and enhancing MUC-2 expression, thereby decreasing LPS transferring from the intestine to serum. MPE also modulated gut microbiota by enriching beneficial bacteria such as *Lactobacillus* and *Ruminococcus* while suppressing potentially harmful taxa. Fecal microbiota transplantation from MPE-treated donors reproduced these protective effects, highlighting the pivotal role of gut microbiota in mediating MPE’s systemic benefits. Collectively, these findings elucidate the mechanisms by which MPE alleviates hepatic oxidative stress and provide a promising method for preventing hepatic oxidative damage by modulating the gut microbiota–gut–liver axis in poultry.

## Data Availability

The original contributions presented in this study are included in the article/Supplementary Material. Further inquiries can be directed to the corresponding author(s).

## Supplementary Materials

The following supporting information can be downloaded at: https://www.mdpi.com/article/10.3390/antiox14091045/s1, Table S1: Ingredients compositions and nutrient levels of the experimental diets (air-dry basis, %). Table S2. The primers for qRT-PCR assays1.
