# Supplementary material for: Mangosteen Pericarp Extract Mitigates Diquat-Induced Hepatic Oxidative Stress by NRF2/HO-1 Activation, Intestinal Barrier Integrity Restoration, and Gut Microbiota Modulation
Source: Antioxidants (Basel). 2025 Aug 25;14(9):1045. doi: 10.3390/antiox14091045 (PMC12466791; doi:10.3390/antiox14091045)
Supplement: Supplementary file 1 [file antioxidants-14-01045-s001.zip › antioxidants-3762277-supplementary.pdf]

Table S1. Ingredients compositions and nutrient levels of the experimental diets (air-dry basis, %).

Items	Basal diet	MPE contained diet
**Ingredients**		
Corn	57.00	57.00
Soybean meal	24.00	24.00
Wheat middling	5.00	5.00
Emulsified fat powder	1.50	1.50
Limestone	9.00	9.00
$CaHPO_4$	1.00	1.00
Salt	0.30	0.30
DL-Methionine	0.20	0.20
Premix[1]	1.75	1.75
Zeolite powder	0.25	-
MPE premix	-	0.25
Total	100.00	100.00
**Nutrient levels[2]**		
Metabolizable energy, MCal/kg	2.63	2.64
Organic matter	84.05	84.04
Crude protein	16.35	16.37
Lysine, %	0.83	0.82
Ash	6.63	6.64
Methionine	0.48	0.48
Cysteine + methionine	0.75	0.76
Calcium	3.61	3.60
Total phosphorus, %	0.67	0.67

[1] The premix provided the following per kg of the diet: vitamin A, 12,500 IU; vitamin D3, 4,000 IU; vitamin K3, 2 mg; thiamine, 1 mg; riboflavin, 8.5 mg; calcium pantothenate, 50 mg; niacin acid, 32.5 mg; pyridoxine, 8 mg; folic acid, 5 mg; B12, 5 mg; choline chloride, 500 mg; iron, 60 mg; copper, 10mg; manganese, 80 mg; zinc 80 mg; iodine 0.3 mg; and selenium 0.3 mg.

[2] Metabolizable energy (ME) in the nutrient levels were calculated values, while the others were measured values. ME values were drawn from the Chinese Feed Composition and Nutritional Value Table (32th edition, 2021), Chinese feed database.

Table S2. The primers for qRT-PCR assays1.

Target genes	Forward primer (5′→3)	Reverse primer (5′→3′)	GenBank accession	Size (bp)	Efficiency (%)
β-actin	GATATTGCTGCGCTCGTTGT	TACCAACCATCACACCCTGAT	NM_205518.2	130	99
TGF-β2	GCTGTACCAGGGTTACGGCA ATG	ACGTCGAAGGAGAGCCACTC ATC	NM_001318456.1	150	98
ACTA-2	ACTGCTGCCTCTTCCTCCTCT C	AGCGGACTCCATGCCAATGA AAG	XM_046920244.1	116	97
HNF4B	GTTTTTGCCGCTTCCCTTGT	CGCCCATTAGCCTCCTCATT	NM_001030576.5	98	98
PDGFRA	CGCGTTATAAAGGAGGAGCT GT	GCCACTGTTGTTCTCCTCGT	NM_204749.3	100	96
IL-6	AACAACCTCAACCTGCCCAA	TTCGTCAGGCATTTCTCCTCG	NM_204628.2	80	98
ZO-1	AACGCAGCTATTATCCGGCA	ATGCTCATAGCGAGGTCTGC	XM_040706827.2	80	99
MUC-2	GCTCACCCTGCATGGATACT	TCATACACAGTCCCTTCGGG	XM_040701654.2	100	99
CAT	CCTGACTATGGCGCACGTAT	CAGACACACGAGAAGTGGCT	NM_001031215.2	106	99
GST	AATTTCCCCTCTTGCAGAGT T	TCACTCCACTTATCAGCAAAC AG	NM_001001777.2	134	94
IL-10	GACGTTCGAGAAGATGGATG A	AGCAGGTACTCCTCGATGTA	NM_001004414.4	87	97
TNF-α	GATCGTGACACGTCTCTGCT	AACCAGCTATGCACCCCAG	NM_204267.2	86	98
NF-κB	TGATCCAGCAGATGGACCGT	AGAAGCACCAGGAAGTCCAC	XM_046939919.1	112	97
KEAP-1	TCAACTGGGTGCAGTACGAC	TCTGCGCCAGGTAATCCTTG	MN416132.1	145	98
NRF-2	CAGGGGTAGCAAGGTATGAG G	TTCCCAGTTCGGTGCAGAAG	NM_205117.2	100	98
HO-1	CCACACAACGCTGAAAGCAT	GATGAAGTACAGGGACGCCG	NM_205344.2	90	99
NQO-1	AACCTCTTTCAACCACGCCA	AAGCACTCGGGGTTCTTGAG	NM_001277619.2	113	97
Occludin-1	TCATCGTCATCCTGCTCTGC	CACGTTCTTCACCCACTCCT	NM_205128.1	146	99
Claudin-5	TGTCAGCCTTCATCGACGTG	TGGAATCGTACACCTTGCACT	NM_204201.2	114	95
SOD1	TGACCTCGGCAATGTGACTG	CATGGTACGGCCAATGATGC	NM_205064.2	104	98
SOD2	TGTTCAAGGATCAGGCTGGG	CCCAGCAATGGAATGAGACC T	NM_204211.2	150	94

[1] Optimal primer concentration for all primer pairs was 200 nM.
